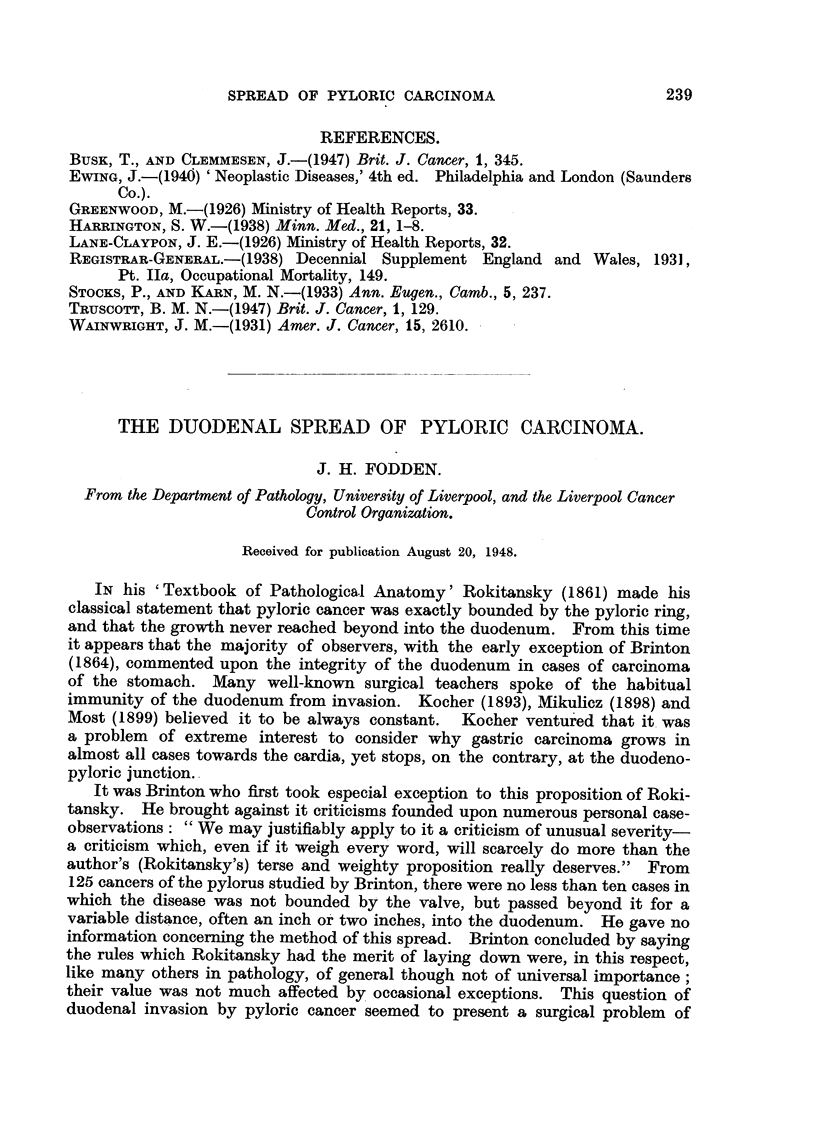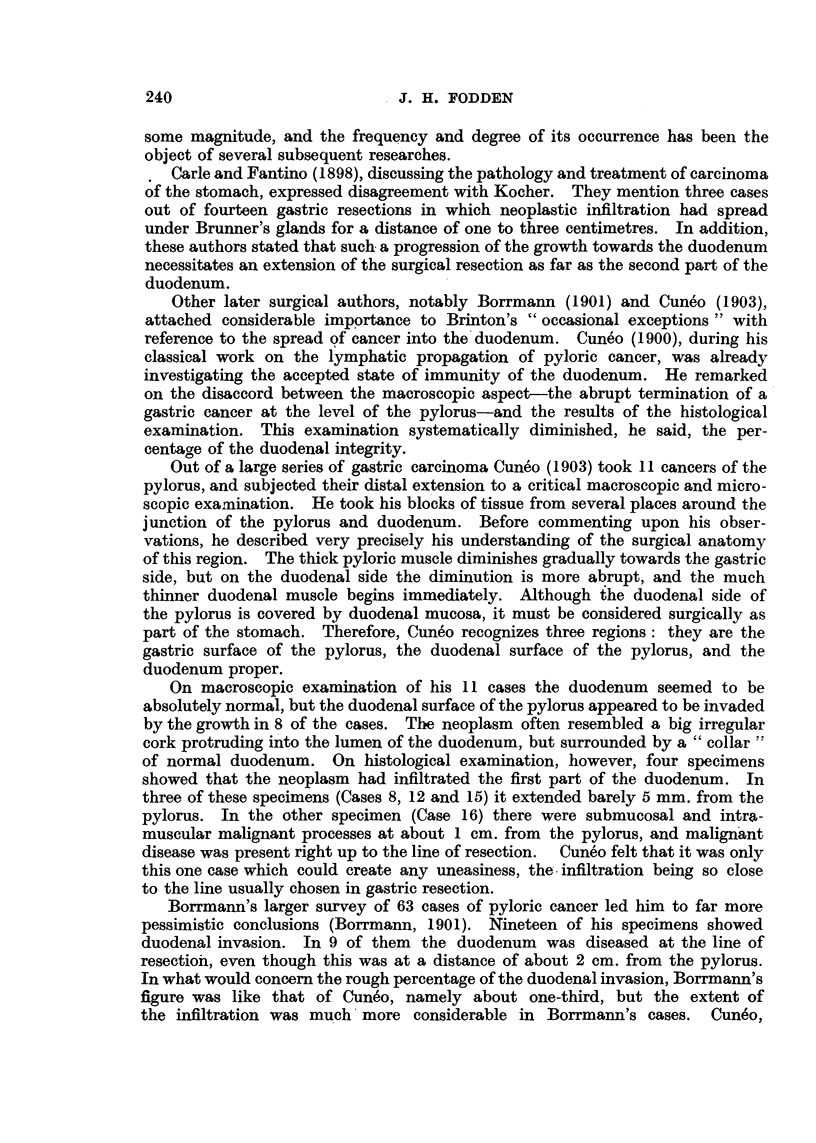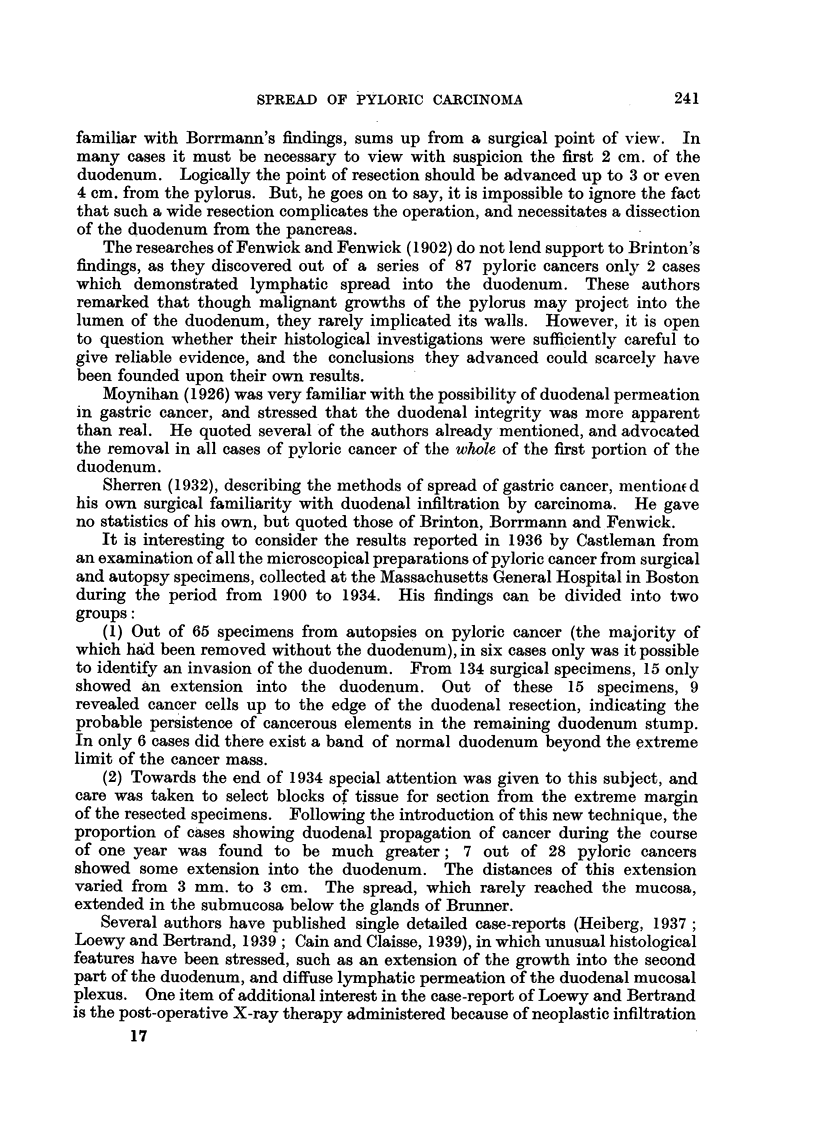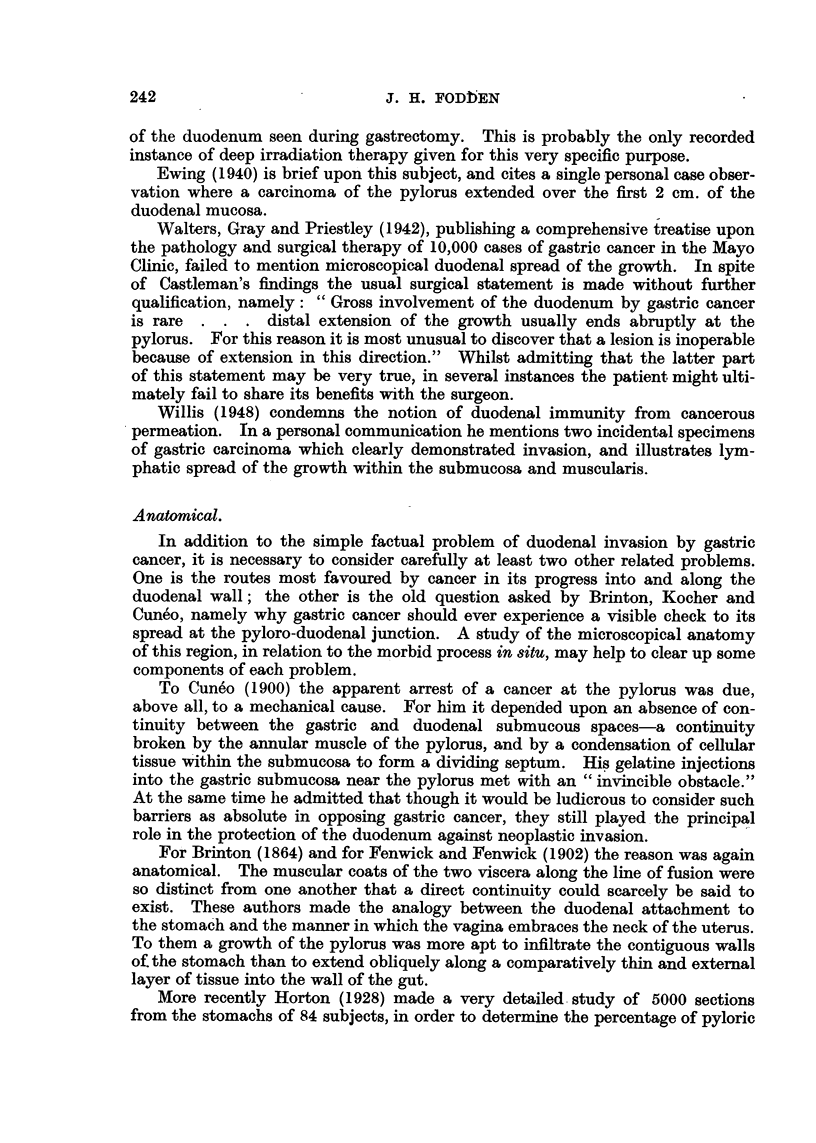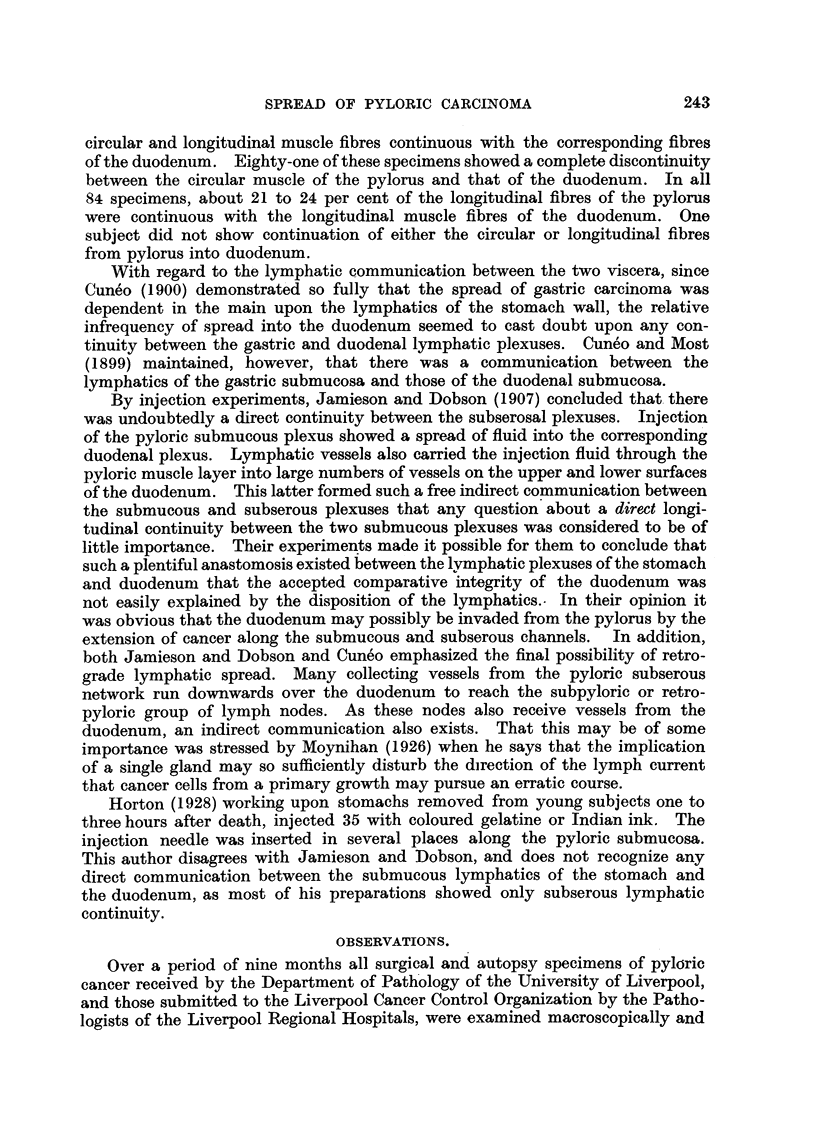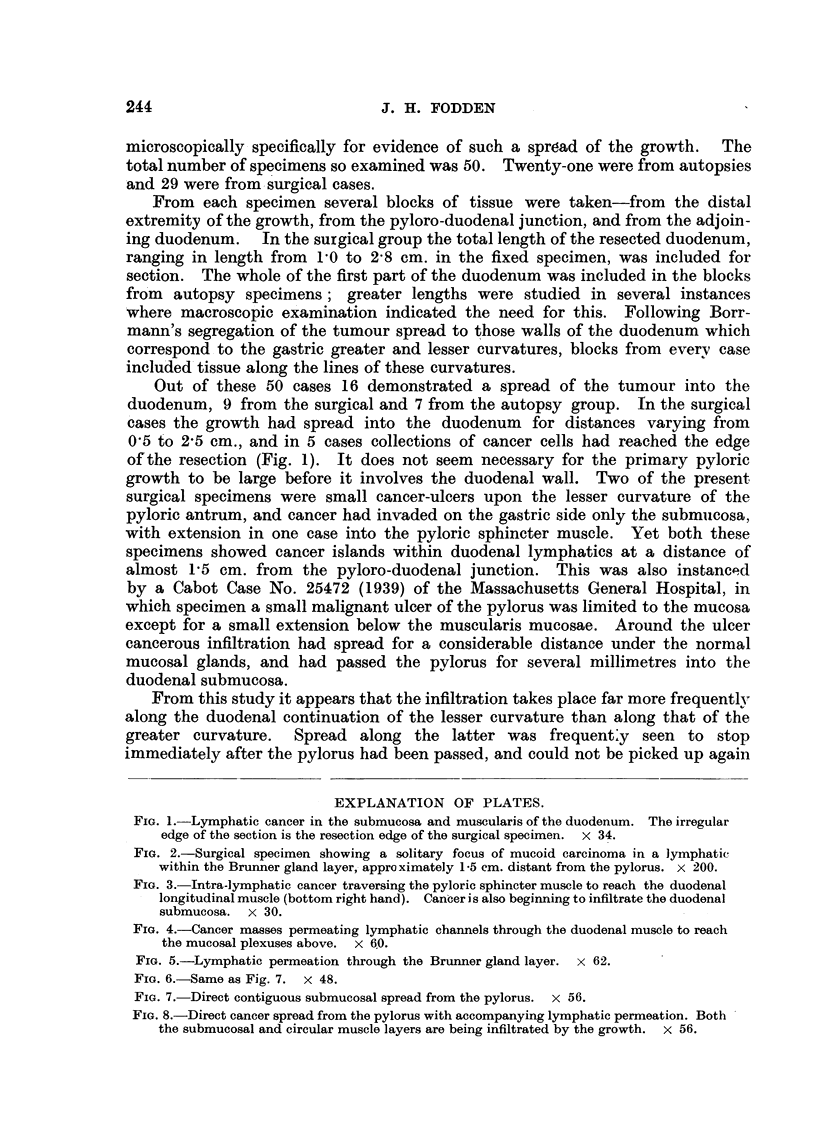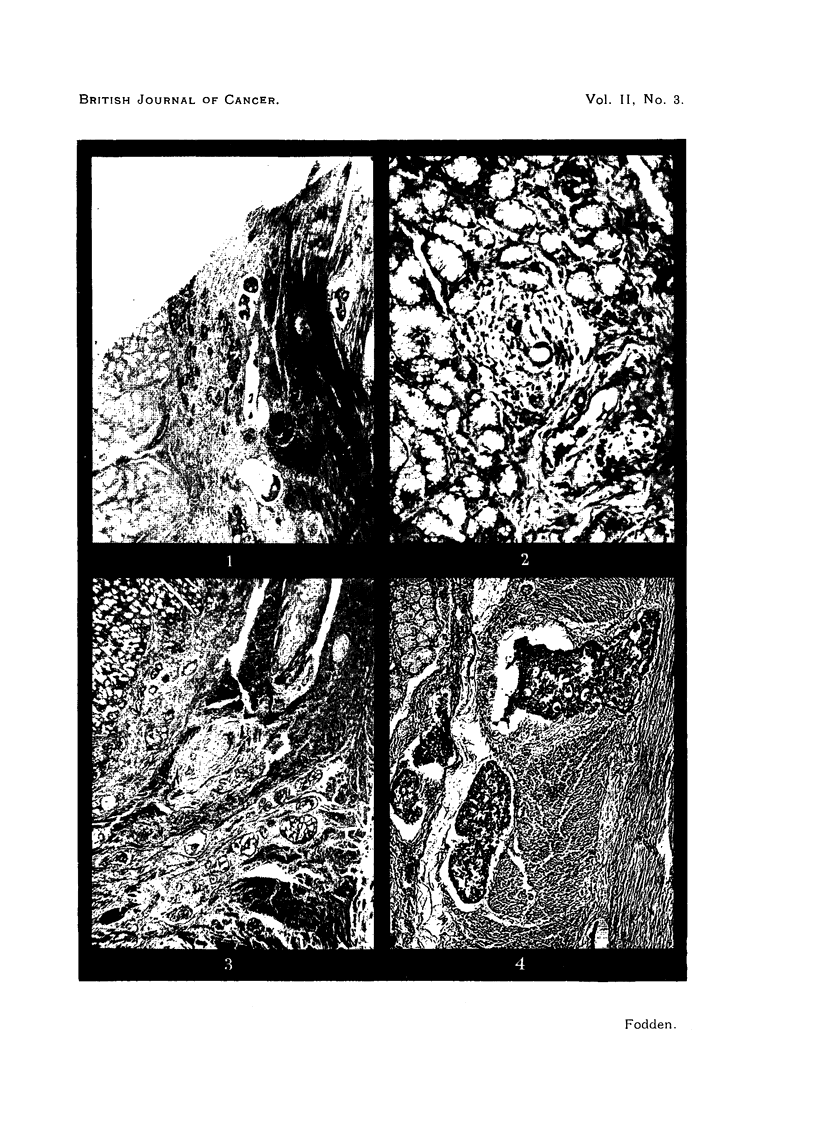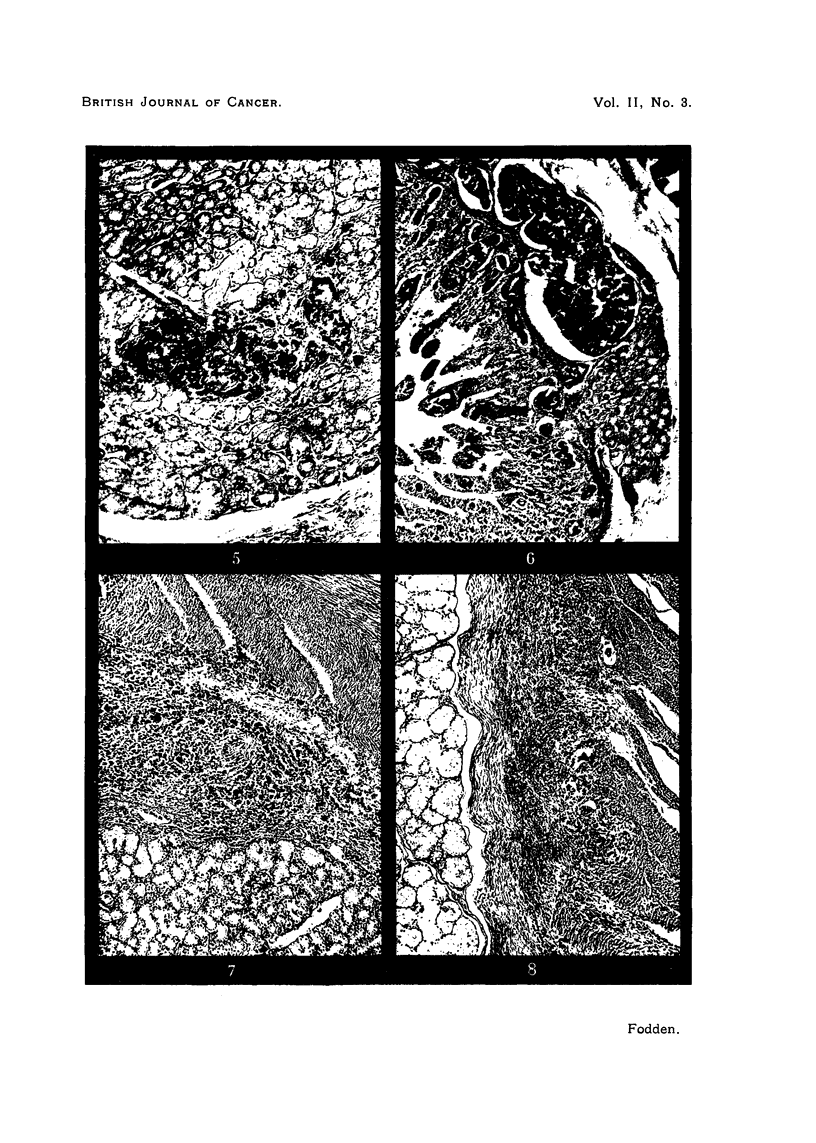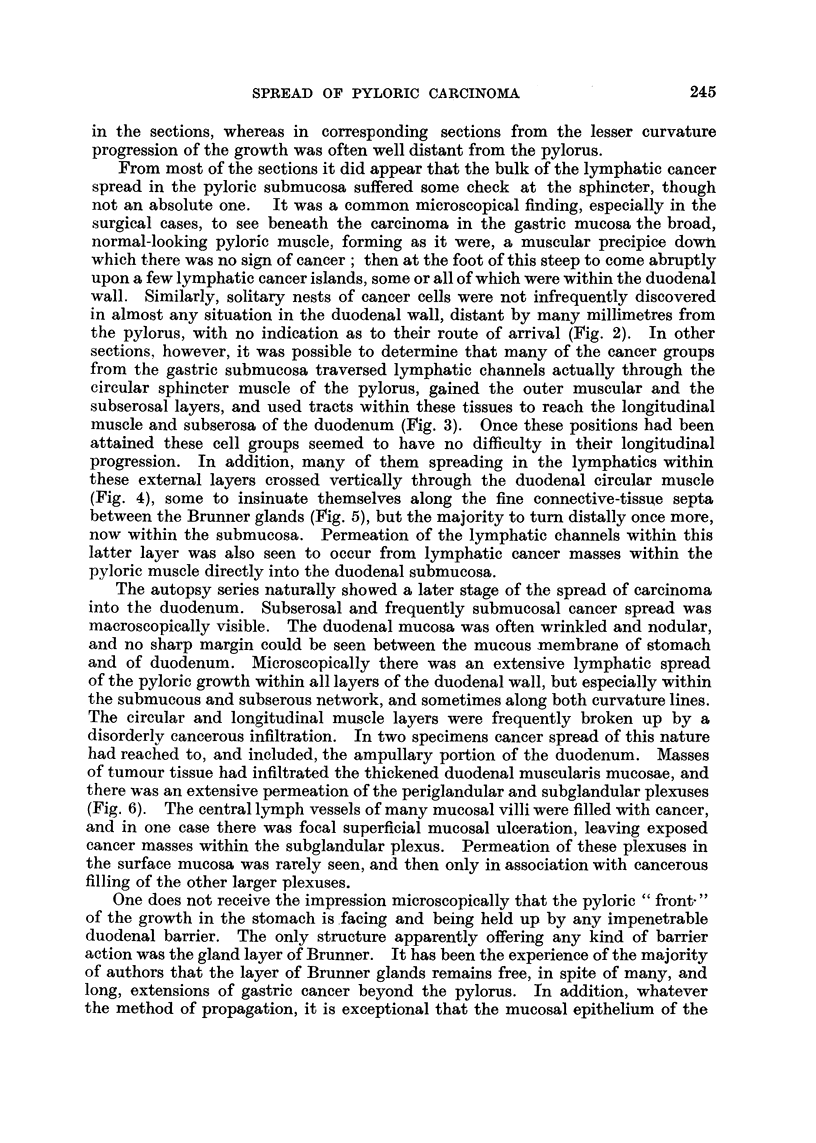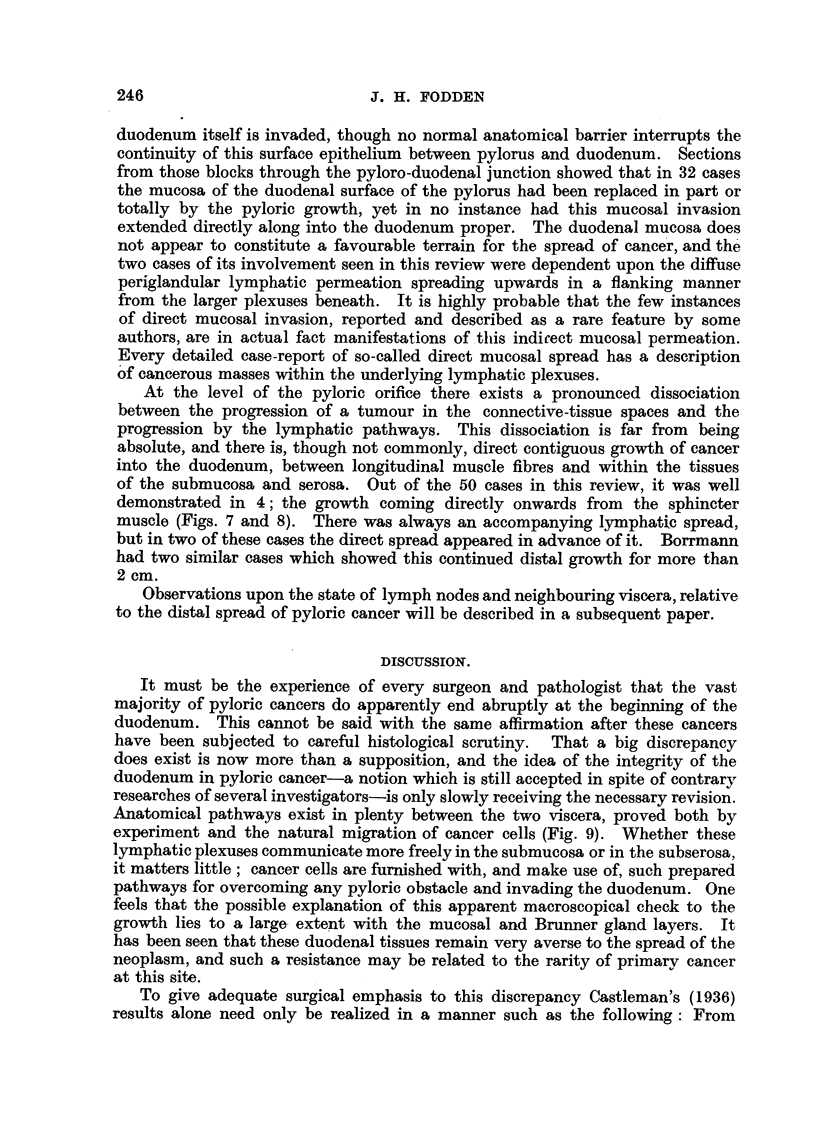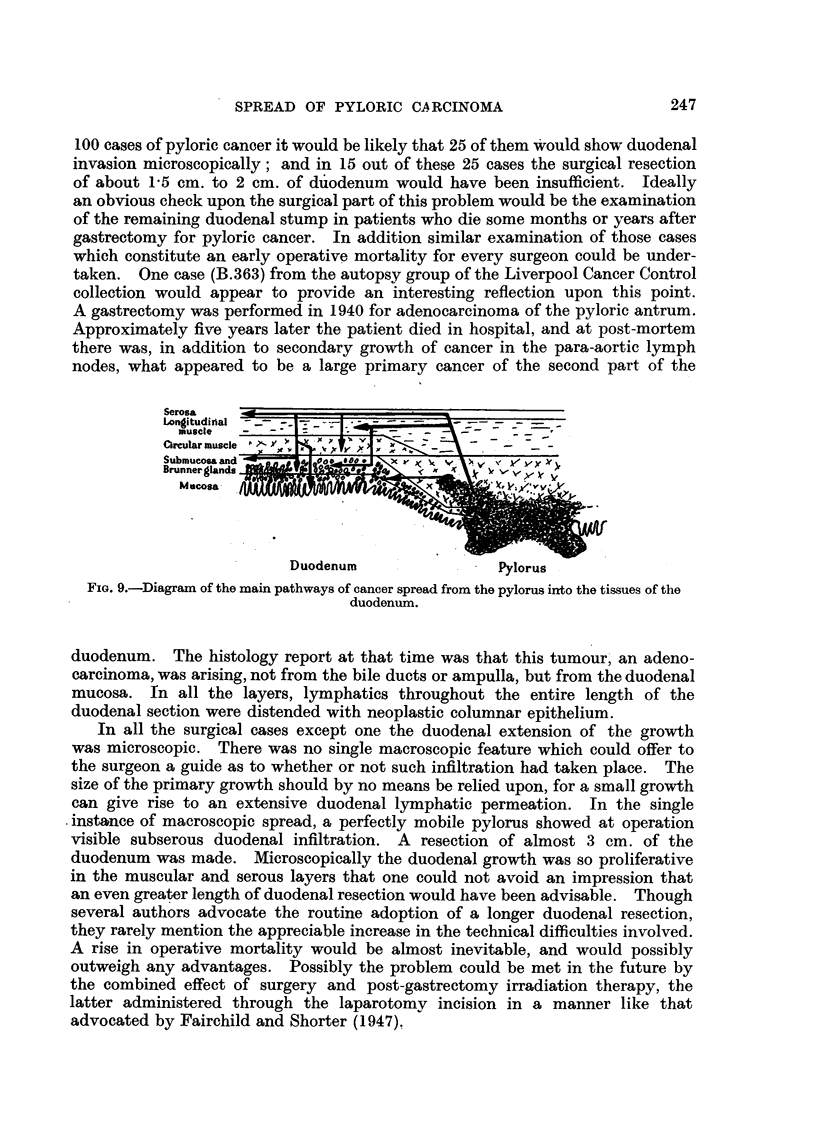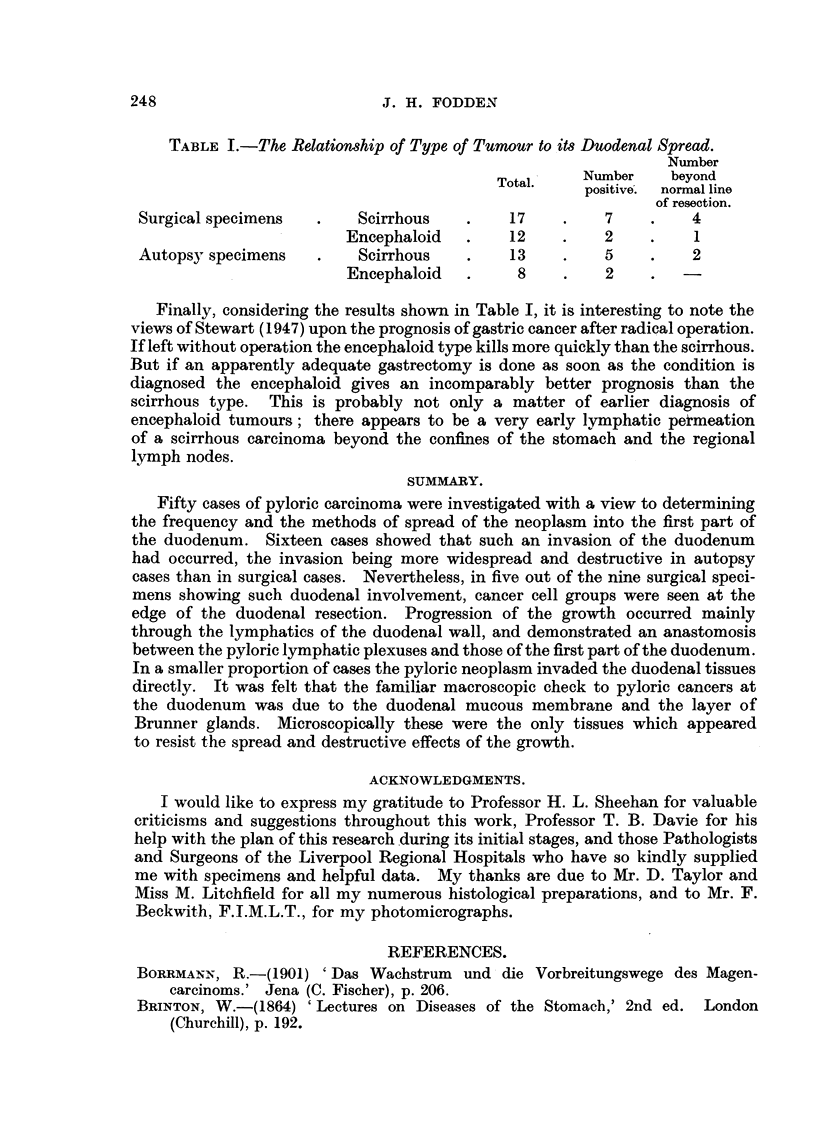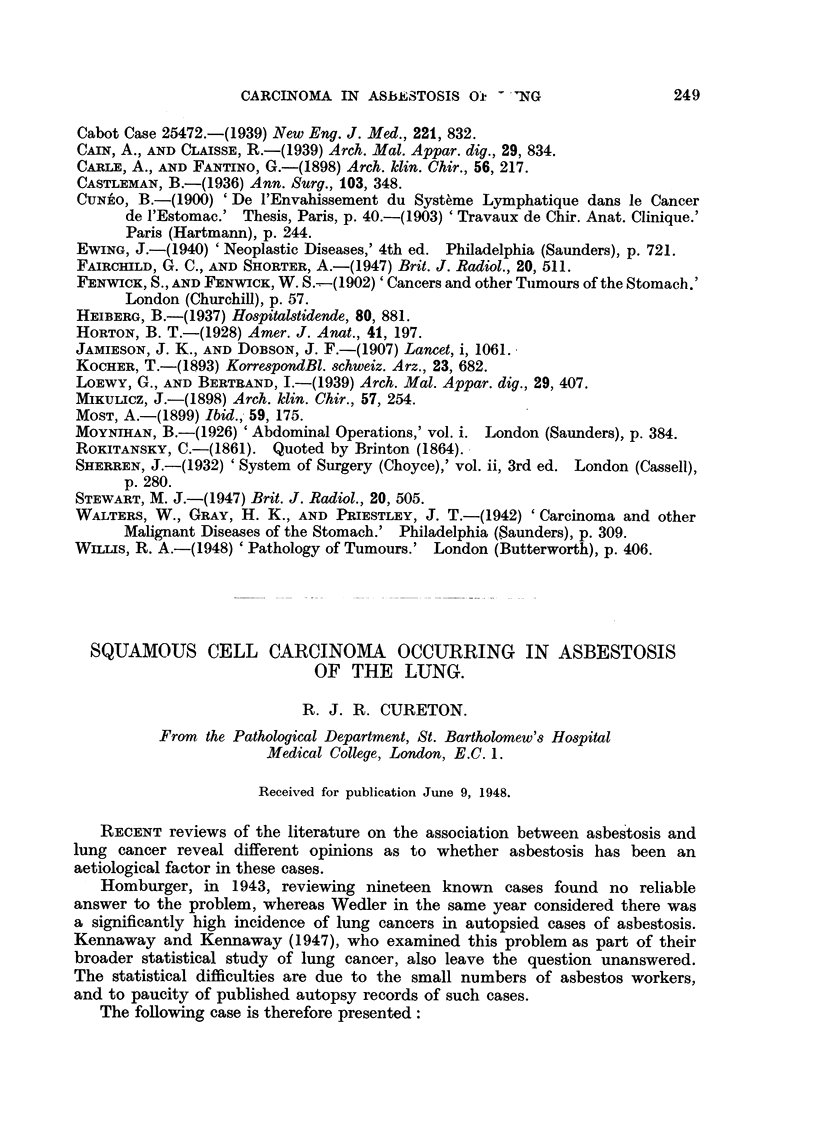# The Duodenal Spread of Pyloric Carcinoma

**DOI:** 10.1038/bjc.1948.28

**Published:** 1948-09

**Authors:** J. H. Fodden

## Abstract

**Images:**


### THE DUODENAL SPREAD OF PYLORIC CARCINOMA.

J. H. FODDEN.

*From the Department of Pathology, University of Liverpool, and the Liverpool Cancer Control Organization.*

Received for publication August 20, 1948.

IN his 'Textbook of Pathological Anatomy' Rokitansky (1861) made his classical statement that pyloric cancer was exactly bounded by the pyloric ring, and that the growth never reached beyond into the duodenum. From this time it appears that the majority of observers, with the early exception of Brinton (1864), commented upon the integrity of the duodenum in cases of carcinoma of the stomach. Many well-known surgical teachers spoke of the habitual immunity of the duodenum from invasion. Kocher (1893), Mikulicz (1898) and Most (1899) believed it to be always constant. Kocher ventured that it was a problem of extreme interest to consider why gastric carcinoma grows in almost all cases towards the cardia, yet stops, on the contrary, at the duodeno-pyloric junction.

It was Brinton who first took especial exception to this proposition of Rokitansky. He brought against it criticisms founded upon numerous personal case-observations : " We may justifiably apply to it a criticism of unusual severity—a criticism which, even if it weigh every word, will scarcely do more than the author's (Rokitansky's) terse and weighty proposition really deserves." From 125 cancers of the pylorus studied by Brinton, there were no less than ten cases in which the disease was not bounded by the valve, but passed beyond it for a variable distance, often an inch or two inches, into the duodenum. He gave no information concerning the method of this spread. Brinton concluded by saying the rules which Rokitansky had the merit of laying down were, in this respect, like many others in pathology, of general though not of universal importance ; their value was not much affected by occasional exceptions. This question of duodenal invasion by pyloric cancer seemed to present a surgical problem of some magnitude, and the frequency and degree of its occurrence has been the object of several subsequent researches.

Carle and Fantino (1898), discussing the pathology and treatment of carcinoma of the stomach, expressed disagreement with Kocher. They mention three cases out of fourteen gastric resections in which neoplastic infiltration had spread under Brunner's glands for a distance of one to three centimetres. In addition, these authors stated that such a progression of the growth towards the duodenum necessitates an extension of the surgical resection as far as the second part of the duodenum.

Other later surgical authors, notably Borrmann (1901) and Cunéo (1903), attached considerable importance to Brinton's "occasional exceptions" with reference to the spread of cancer into the duodenum. Cunéo (1900), during his classical work on the lymphatic propagation of pyloric cancer, was already investigating the accepted state of immunity of the duodenum. He remarked on the disaccord between the macroscopic aspect—the abrupt termination of a gastric cancer at the level of the pylorus—and the results of the histological examination. This examination systematically diminished, he said, the percentage of the duodenal integrity.

Out of a large series of gastric carcinoma Cunéo (1903) took 11 cancers of the pylorus, and subjected their distal extension to a critical macroscopic and microscopic examination. He took his blocks of tissue from several places around the junction of the pylorus and duodenum. Before commenting upon his observations, he described very precisely his understanding of the surgical anatomy of this region. The thick pyloric muscle diminishes gradually towards the gastric side, but on the duodenal side the diminution is more abrupt, and the much thinner duodenal muscle begins immediately. Although the duodenal side of the pylorus is covered by duodenal mucosa, it must be considered surgically as part of the stomach. Therefore, Cunéo recognizes three regions: they are the gastric surface of the pylorus, the duodenal surface of the pylorus, and the duodenum proper.

On macroscopic examination of his 11 cases the duodenum seemed to be absolutely normal, but the duodenal surface of the pylorus appeared to be invaded by the growth in 8 of the cases. The neoplasm often resembled a big irregular cork protruding into the lumen of the duodenum, but surrounded by a "collar" of normal duodenum. On histological examination, however, four specimens showed that the neoplasm had infiltrated the first part of the duodenum. In three of these specimens (Cases 8, 12 and 15) it extended barely 5 mm. from the pylorus. In the other specimen (Case 16) there were submucosal and intramuscular malignant processes at about 1 cm. from the pylorus, and malignant disease was present right up to the line of resection. Cunéo felt that it was only this one case which could create any uneasiness, the infiltration being so close to the line usually chosen in gastric resection.

Borrmann's larger survey of 63 cases of pyloric cancer led him to far more pessimistic conclusions (Borrmann, 1901). Nineteen of his specimens showed duodenal invasion. In 9 of them the duodenum was diseased at the line of resection, even though this was at a distance of about 2 cm. from the pylorus. In what would concern the rough percentage of the duodenal invasion, Borrmann's figure was like that of Cunéo, namely about one-third, but the extent of the infiltration was much more considerable in Borrmann's cases. Cunéo, familiar with Borrmann's findings, sums up from a surgical point of view. In many cases it must be necessary to view with suspicion the first 2 cm. of the duodenum. Logically the point of resection should be advanced up to 3 or even 4 cm. from the pylorus. But, he goes on to say, it is impossible to ignore the fact that such a wide resection complicates the operation, and necessitates a dissection of the duodenum from the pancreas.

The researches of Fenwick and Fenwick (1902) do not lend support to Brinton's findings, as they discovered out of a series of 87 pyloric cancers only 2 cases which demonstrated lymphatic spread into the duodenum. These authors remarked that though malignant growths of the pylorus may project into the lumen of the duodenum, they rarely implicated its walls. However, it is open to question whether their histological investigations were sufficiently careful to give reliable evidence, and the conclusions they advanced could scarcely have been founded upon their own results.

Moynihan (1926) was very familiar with the possibility of duodenal permeation in gastric cancer, and stressed that the duodenal integrity was more apparent than real. He quoted several of the authors already mentioned, and advocated the removal in all cases of pyloric cancer of the *whole* of the first portion of the duodenum.

Sherren (1932), describing the methods of spread of gastric cancer, mentioned his own surgical familiarity with duodenal infiltration by carcinoma. He gave no statistics of his own, but quoted those of Brinton, Borrmann and Fenwick.

It is interesting to consider the results reported in 1936 by Castleman from an examination of all the microscopical preparations of pyloric cancer from surgical and autopsy specimens, collected at the Massachusetts General Hospital in Boston during the period from 1900 to 1934. His findings can be divided into two groups:

(1) Out of 65 specimens from autopsies on pyloric cancer (the majority of which had been removed without the duodenum), in six cases only was it possible to identify an invasion of the duodenum. From 134 surgical specimens, 15 only showed an extension into the duodenum. Out of these 15 specimens, 9 revealed cancer cells up to the edge of the duodenal resection, indicating the probable persistence of cancerous elements in the remaining duodenum stump. In only 6 cases did there exist a band of normal duodenum beyond the extreme limit of the cancer mass.

(2) Towards the end of 1934 special attention was given to this subject, and care was taken to select blocks of tissue for section from the extreme margin of the resected specimens. Following the introduction of this new technique, the proportion of cases showing duodenal propagation of cancer during the course of one year was found to be much greater; 7 out of 28 pyloric cancers showed some extension into the duodenum. The distances of this extension varied from 3 mm. to 3 cm. The spread, which rarely reached the mucosa, extended in the submucosa below the glands of Brunner.

Several authors have published single detailed case-reports (Heiberg, 1937; Loewy and Bertrand, 1939; Cain and Claisse, 1939), in which unusual histological features have been stressed, such as an extension of the growth into the second part of the duodenum, and diffuse lymphatic permeation of the duodenal mucosal plexus. One item of additional interest in the case-report of Loewy and Bertrand is the post-operative X-ray therapy administered because of neoplastic infiltration of the duodenum seen during gastrectomy. This is probably the only recorded instance of deep irradiation therapy given for this very specific purpose.

Ewing (1940) is brief upon this subject, and cites a single personal case observation where a carcinoma of the pylorus extended over the first 2 cm. of the duodenal mucosa.

Walters, Gray and Priestley (1942), publishing a comprehensive treatise upon the pathology and surgical therapy of 10,000 cases of gastric cancer in the Mayo Clinic, failed to mention microscopical duodenal spread of the growth. In spite of Castleman's findings the usual surgical statement is made without further qualification, namely: "Gross involvement of the duodenum by gastric cancer is rare . . . distal extension of the growth usually ends abruptly at the pylorus. For this reason it is most unusual to discover that a lesion is inoperable because of extension in this direction." Whilst admitting that the latter part of this statement may be very true, in several instances the patient might ultimately fail to share its benefits with the surgeon.

Willis (1948) condemns the notion of duodenal immunity from cancerous permeation. In a personal communication he mentions two incidental specimens of gastric carcinoma which clearly demonstrated invasion, and illustrates lymphatic spread of the growth within the submucosa and muscularis.

*Anatomical.*

In addition to the simple factual problem of duodenal invasion by gastric cancer, it is necessary to consider carefully at least two other related problems. One is the routes most favoured by cancer in its progress into and along the duodenal wall; the other is the old question asked by Brinton, Kocher and Cunéo, namely why gastric cancer should ever experience a visible check to its spread at the pyloro-duodenal junction. A study of the microscopical anatomy of this region, in relation to the morbid process *in situ*, may help to clear up some components of each problem.

To Cunéo (1900) the apparent arrest of a cancer at the pylorus was due, above all, to a mechanical cause. For him it depended upon an absence of continuity between the gastric and duodenal submucous spaces—a continuity broken by the annular muscle of the pylorus, and by a condensation of cellular tissue within the submucosa to form a dividing septum. His gelatine injections into the gastric submucosa near the pylorus met with an "invincible obstacle." At the same time he admitted that though it would be ludicrous to consider such barriers as absolute in opposing gastric cancer, they still played the principal role in the protection of the duodenum against neoplastic invasion.

For Brinton (1864) and for Fenwick and Fenwick (1902) the reason was again anatomical. The muscular coats of the two viscera along the line of fusion were so distinct from one another that a direct continuity could scarcely be said to exist. These authors made the analogy between the duodenal attachment to the stomach and the manner in which the vagina embraces the neck of the uterus. To them a growth of the pylorus was more apt to infiltrate the contiguous walls of the stomach than to extend obliquely along a comparatively thin and external layer of tissue into the wall of the gut.

More recently Horton (1928) made a very detailed study of 5000 sections from the stomachs of 84 subjects, in order to determine the percentage of pyloric circular and longitudinal muscle fibres continuous with the corresponding fibres of the duodenum. Eighty-one of these specimens showed a complete discontinuity between the circular muscle of the pylorus and that of the duodenum. In all 84 specimens, about 21 to 24 per cent of the longitudinal fibres of the pylorus were continuous with the longitudinal muscle fibres of the duodenum. One subject did not show continuation of either the circular or longitudinal fibres from pylorus into duodenum.

With regard to the lymphatic communication between the two viscera, since Cunéo (1900) demonstrated so fully that the spread of gastric carcinoma was dependent in the main upon the lymphatics of the stomach wall, the relative infrequency of spread into the duodenum seemed to cast doubt upon any continuity between the gastric and duodenal lymphatic plexuses. Cunéo and Most (1899) maintained, however, that there was a communication between the lymphatics of the gastric submucosa and those of the duodenal submucosa.

By injection experiments, Jamieson and Dobson (1907) concluded that there was undoubtedly a direct continuity between the subserosal plexuses. Injection of the pyloric submucous plexus showed a spread of fluid into the corresponding duodenal plexus. Lymphatic vessels also carried the injection fluid through the pyloric muscle layer into large numbers of vessels on the upper and lower surfaces of the duodenum. This latter formed such a free indirect communication between the submucous and subserous plexuses that any question about a *direct* longitudinal continuity between the two submucous plexuses was considered to be of little importance. Their experiments made it possible for them to conclude that such a plentiful anastomosis existed between the lymphatic plexuses of the stomach and duodenum that the accepted comparative integrity of the duodenum was not easily explained by the disposition of the lymphatics. In their opinion it was obvious that the duodenum may possibly be invaded from the pylorus by the extension of cancer along the submucous and subserous channels. In addition, both Jamieson and Dobson and Cunéo emphasized the final possibility of retrograde lymphatic spread. Many collecting vessels from the pyloric subserous network run downwards over the duodenum to reach the subpyloric or retropyloric group of lymph nodes. As these nodes also receive vessels from the duodenum, an indirect communication also exists. That this may be of some importance was stressed by Moynihan (1926) when he says that the implication of a single gland may so sufficiently disturb the direction of the lymph current that cancer cells from a primary growth may pursue an erratic course.

Horton (1928) working upon stomachs removed from young subjects one to three hours after death, injected 35 with coloured gelatine or Indian ink. The injection needle was inserted in several places along the pyloric submucosa. This author disagrees with Jamieson and Dobson, and does not recognize any direct communication between the submucous lymphatics of the stomach and the duodenum, as most of his preparations showed only subserous lymphatic continuity.

### OBSERVATIONS.

Over a period of nine months all surgical and autopsy specimens of pyloric cancer received by the Department of Pathology of the University of Liverpool, and those submitted to the Liverpool Cancer Control Organization by the Pathologists of the Liverpool Regional Hospitals, were examined macroscopically and microscopically specifically for evidence of such a spread of the growth. The total number of specimens so examined was 50. Twenty-one were from autopsies and 29 were from surgical cases.

From each specimen several blocks of tissue were taken—from the distal extremity of the growth, from the pyloro-duodenal junction, and from the adjoining duodenum. In the surgical group the total length of the resected duodenum, ranging in length from 1·0 to 2·8 cm. in the fixed specimen, was included for section. The whole of the first part of the duodenum was included in the blocks from autopsy specimens; greater lengths were studied in several instances where macroscopic examination indicated the need for this. Following Borrmann's segregation of the tumour spread to those walls of the duodenum which correspond to the gastric greater and lesser curvatures, blocks from every case included tissue along the lines of these curvatures.

Out of these 50 cases 16 demonstrated a spread of the tumour into the duodenum, 9 from the surgical and 7 from the autopsy group. In the surgical cases the growth had spread into the duodenum for distances varying from 0·5 to 2·5 cm., and in 5 cases collections of cancer cells had reached the edge of the resection (Fig. 1). It does not seem necessary for the primary pyloric growth to be large before it involves the duodenal wall. Two of the present surgical specimens were small cancer-ulcers upon the lesser curvature of the pyloric antrum, and cancer had invaded on the gastric side only the submucosa, with extension in one case into the pyloric sphincter muscle. Yet both these specimens showed cancer islands within duodenal lymphatics at a distance of almost 1·5 cm. from the pyloro-duodenal junction. This was also instanced by a Cabot Case No. 25472 (1939) of the Massachusetts General Hospital, in which specimen a small malignant ulcer of the pylorus was limited to the mucosa except for a small extension below the muscularis mucosae. Around the ulcer cancerous infiltration had spread for a considerable distance under the normal mucosal glands, and had passed the pylorus for several millimetres into the duodenal submucosa.

From this study it appears that the infiltration takes place far more frequently along the duodenal continuation of the lesser curvature than along that of the greater curvature. Spread along the latter was frequently seen to stop immediately after the pylorus had been passed, and could not be picked up again

---

EXPLANATION OF PLATES.

FIG. 1.—Lymphatic cancer in the submucosa and muscularis of the duodenum. The irregular edge of the section is the resection edge of the surgical specimen. × 34.

FIG. 2.—Surgical specimen showing a solitary focus of mucoid carcinoma in a lymphatic within the Brunner gland layer, approximately 1·5 cm. distant from the pylorus. × 200.

FIG. 3.—Intra-lymphatic cancer traversing the pyloric sphincter muscle to reach the duodenal longitudinal muscle (bottom right hand). Cancer is also beginning to infiltrate the duodenal submucosa. × 30.

FIG. 4.—Cancer masses permeating lymphatic channels through the duodenal muscle to reach the mucosal plexuses above. × 60.

FIG. 5.—Lymphatic permeation through the Brunner gland layer. × 62.

FIG. 6.—Same as Fig. 7. × 48.

FIG. 7.—Direct contiguous submucosal spread from the pylorus. × 56.

FIG. 8.—Direct cancer spread from the pylorus with accompanying lymphatic permeation. Both the submucosal and circular muscle layers are being infiltrated by the growth. × 56.

1

2

3

4

Fodden.

5

6

7

8

Fodden.

in the sections, whereas in corresponding sections from the lesser curvature progression of the growth was often well distant from the pylorus.

From most of the sections it did appear that the bulk of the lymphatic cancer spread in the pyloric submucosa suffered some check at the sphincter, though not an absolute one. It was a common microscopical finding, especially in the surgical cases, to see beneath the carcinoma in the gastric mucosa the broad, normal-looking pyloric muscle, forming as it were, a muscular precipice down which there was no sign of cancer ; then at the foot of this steep to come abruptly upon a few lymphatic cancer islands, some or all of which were within the duodenal wall. Similarly, solitary nests of cancer cells were not infrequently discovered in almost any situation in the duodenal wall, distant by many millimetres from the pylorus, with no indication as to their route of arrival (Fig. 2). In other sections, however, it was possible to determine that many of the cancer groups from the gastric submucosa traversed lymphatic channels actually through the circular sphincter muscle of the pylorus, gained the outer muscular and the subserosal layers, and used tracts within these tissues to reach the longitudinal muscle and subserosa of the duodenum (Fig. 3). Once these positions had been attained these cell groups seemed to have no difficulty in their longitudinal progression. In addition, many of them spreading in the lymphatics within these external layers crossed vertically through the duodenal circular muscle (Fig. 4), some to insinuate themselves along the fine connective-tissue septa between the Brunner glands (Fig. 5), but the majority to turn distally once more, now within the submucosa. Permeation of the lymphatic channels within this latter layer was also seen to occur from lymphatic cancer masses within the pyloric muscle directly into the duodenal submucosa.

The autopsy series naturally showed a later stage of the spread of carcinoma into the duodenum. Subserosal and frequently submucosal cancer spread was macroscopically visible. The duodenal mucosa was often wrinkled and nodular, and no sharp margin could be seen between the mucous membrane of stomach and of duodenum. Microscopically there was an extensive lymphatic spread of the pyloric growth within all layers of the duodenal wall, but especially within the submucous and subserous network, and sometimes along both curvature lines. The circular and longitudinal muscle layers were frequently broken up by a disorderly cancerous infiltration. In two specimens cancer spread of this nature had reached to, and included, the ampullary portion of the duodenum. Masses of tumour tissue had infiltrated the thickened duodenal muscularis mucosae, and there was an extensive permeation of the periglandular and subglandular plexuses (Fig. 6). The central lymph vessels of many mucosal villi were filled with cancer, and in one case there was focal superficial mucosal ulceration, leaving exposed cancer masses within the subglandular plexus. Permeation of these plexuses in the surface mucosa was rarely seen, and then only in association with cancerous filling of the other larger plexuses.

One does not receive the impression microscopically that the pyloric " front " of the growth in the stomach is facing and being held up by any impenetrable duodenal barrier. The only structure apparently offering any kind of barrier action was the gland layer of Brunner. It has been the experience of the majority of authors that the layer of Brunner glands remains free, in spite of many, and long, extensions of gastric cancer beyond the pylorus. In addition, whatever the method of propagation, it is exceptional that the mucosal epithelium of the duodenum itself is invaded, though no normal anatomical barrier interrupts the continuity of this surface epithelium between pylorus and duodenum. Sections from those blocks through the pyloro-duodenal junction showed that in 32 cases the mucosa of the duodenal surface of the pylorus had been replaced in part or totally by the pyloric growth, yet in no instance had this mucosal invasion extended directly along into the duodenum proper. The duodenal mucosa does not appear to constitute a favourable terrain for the spread of cancer, and the two cases of its involvement seen in this review were dependent upon the diffuse periglandular lymphatic permeation spreading upwards in a flanking manner from the larger plexuses beneath. It is highly probable that the few instances of direct mucosal invasion, reported and described as a rare feature by some authors, are in actual fact manifestations of this indirect mucosal permeation. Every detailed case-report of so-called direct mucosal spread has a description of cancerous masses within the underlying lymphatic plexuses.

At the level of the pyloric orifice there exists a pronounced dissociation between the progression of a tumour in the connective-tissue spaces and the progression by the lymphatic pathways. This dissociation is far from being absolute, and there is, though not commonly, direct contiguous growth of cancer into the duodenum, between longitudinal muscle fibres and within the tissues of the submucosa and serosa. Out of the 50 cases in this review, it was well demonstrated in 4; the growth coming directly onwards from the sphincter muscle (Figs. 7 and 8). There was always an accompanying lymphatic spread, but in two of these cases the direct spread appeared in advance of it. Borrmann had two similar cases which showed this continued distal growth for more than 2 cm.

Observations upon the state of lymph nodes and neighbouring viscera, relative to the distal spread of pyloric cancer will be described in a subsequent paper.

### DISCUSSION.

It must be the experience of every surgeon and pathologist that the vast majority of pyloric cancers do apparently end abruptly at the beginning of the duodenum. This cannot be said with the same affirmation after these cancers have been subjected to careful histological scrutiny. That a big discrepancy does exist is now more than a supposition, and the idea of the integrity of the duodenum in pyloric cancer—a notion which is still accepted in spite of contrary researches of several investigators—is only slowly receiving the necessary revision. Anatomical pathways exist in plenty between the two viscera, proved both by experiment and the natural migration of cancer cells (Fig. 9). Whether these lymphatic plexuses communicate more freely in the submucosa or in the subserosa, it matters little ; cancer cells are furnished with, and make use of, such prepared pathways for overcoming any pyloric obstacle and invading the duodenum. One feels that the possible explanation of this apparent macroscopical check to the growth lies to a large extent with the mucosal and Brunner gland layers. It has been seen that these duodenal tissues remain very averse to the spread of the neoplasm, and such a resistance may be related to the rarity of primary cancer at this site.

To give adequate surgical emphasis to this discrepancy Castleman's (1936) results alone need only be realized in a manner such as the following : From

100 cases of pyloric cancer it would be likely that 25 of them would show duodenal invasion microscopically; and in 15 out of these 25 cases the surgical resection of about 1·5 cm. to 2 cm. of duodenum would have been insufficient. Ideally an obvious check upon the surgical part of this problem would be the examination of the remaining duodenal stump in patients who die some months or years after gastrectomy for pyloric cancer. In addition similar examination of those cases which constitute an early operative mortality for every surgeon could be undertaken. One case (B.363) from the autopsy group of the Liverpool Cancer Control collection would appear to provide an interesting reflection upon this point. A gastrectomy was performed in 1940 for adenocarcinoma of the pyloric antrum. Approximately five years later the patient died in hospital, and at post-mortem there was, in addition to secondary growth of cancer in the para-aortic lymph nodes, what appeared to be a large primary cancer of the second part of the

FIG. 9.—Diagram of the main pathways of cancer spread from the pylorus into the tissues of the duodenum.

duodenum. The histology report at that time was that this tumour, an adenocarcinoma, was arising, not from the bile ducts or ampulla, but from the duodenal mucosa. In all the layers, lymphatics throughout the entire length of the duodenal section were distended with neoplastic columnar epithelium.

In all the surgical cases except one the duodenal extension of the growth was microscopic. There was no single macroscopic feature which could offer to the surgeon a guide as to whether or not such infiltration had taken place. The size of the primary growth should by no means be relied upon, for a small growth can give rise to an extensive duodenal lymphatic permeation. In the single instance of macroscopic spread, a perfectly mobile pylorus showed at operation visible subserous duodenal infiltration. A resection of almost 3 cm. of the duodenum was made. Microscopically the duodenal growth was so proliferative in the muscular and serous layers that one could not avoid an impression that an even greater length of duodenal resection would have been advisable. Though several authors advocate the routine adoption of a longer duodenal resection, they rarely mention the appreciable increase in the technical difficulties involved. A rise in operative mortality would be almost inevitable, and would possibly outweigh any advantages. Possibly the problem could be met in the future by the combined effect of surgery and post-gastrectomy irradiation therapy, the latter administered through the laparotomy incision in a manner like that advocated by Fairchild and Shorter (1947).

TABLE I.—*The Relationship of Type of Tumour to its Duodenal Spread.*

| | | Total. | Number positive. | Number beyond normal line of resection. |
|---|---|---|---|---|
| Surgical specimens | Scirrhous | 17 | 7 | 4 |
| | Encephaloid | 12 | 2 | 1 |
| Autopsy specimens | Scirrhous | 13 | 5 | 2 |
| | Encephaloid | 8 | 2 | — |

Finally, considering the results shown in Table I, it is interesting to note the views of Stewart (1947) upon the prognosis of gastric cancer after radical operation. If left without operation the encephaloid type kills more quickly than the scirrhous. But if an apparently adequate gastrectomy is done as soon as the condition is diagnosed the encephaloid gives an incomparably better prognosis than the scirrhous type. This is probably not only a matter of earlier diagnosis of encephaloid tumours; there appears to be a very early lymphatic permeation of a scirrhous carcinoma beyond the confines of the stomach and the regional lymph nodes.

### SUMMARY.

Fifty cases of pyloric carcinoma were investigated with a view to determining the frequency and the methods of spread of the neoplasm into the first part of the duodenum. Sixteen cases showed that such an invasion of the duodenum had occurred, the invasion being more widespread and destructive in autopsy cases than in surgical cases. Nevertheless, in five out of the nine surgical specimens showing such duodenal involvement, cancer cell groups were seen at the edge of the duodenal resection. Progression of the growth occurred mainly through the lymphatics of the duodenal wall, and demonstrated an anastomosis between the pyloric lymphatic plexuses and those of the first part of the duodenum. In a smaller proportion of cases the pyloric neoplasm invaded the duodenal tissues directly. It was felt that the familiar macroscopic check to pyloric cancers at the duodenum was due to the duodenal mucous membrane and the layer of Brunner glands. Microscopically these were the only tissues which appeared to resist the spread and destructive effects of the growth.

### ACKNOWLEDGMENTS.

I would like to express my gratitude to Professor H. L. Sheehan for valuable criticisms and suggestions throughout this work, Professor T. B. Davie for his help with the plan of this research during its initial stages, and those Pathologists and Surgeons of the Liverpool Regional Hospitals who have so kindly supplied me with specimens and helpful data. My thanks are due to Mr. D. Taylor and Miss M. Litchfield for all my numerous histological preparations, and to Mr. F. Beckwith, F.I.M.L.T., for my photomicrographs.

### REFERENCES.

BORRMANN, R.—(1901) 'Das Wachstrum und die Vorbreitungswege des Magen-carcinoms.' Jena (C. Fischer), p. 206.

BRINTON, W.—(1864) 'Lectures on Diseases of the Stomach,' 2nd ed. London (Churchill), p. 192.

Cabot Case 25472.—(1939) *New Eng. J. Med.*, **221**, 832.
Cain, A., and Claisse, R.—(1939) *Arch. Mal. Appar. dig.*, **29**, 834.
Carle, A., and Fantino, G.—(1898) *Arch. klin. Chir.*, **56**, 217.
Castleman, B.—(1936) *Ann. Surg.*, **103**, 348.
Cunéo, B.—(1900) 'De l'Envahissement du Système Lymphatique dans le Cancer de l'Estomac.' Thesis, Paris, p. 40.—(1903) 'Travaux de Chir. Anat. Clinique.' Paris (Hartmann), p. 244.
Ewing, J.—(1940) 'Neoplastic Diseases,' 4th ed. Philadelphia (Saunders), p. 721.
Fairchild, G. C., and Shorter, A.—(1947) *Brit. J. Radiol.*, **20**, 511.
Fenwick, S., and Fenwick, W. S.—(1902) 'Cancers and other Tumours of the Stomach.' London (Churchill), p. 57.
Heiberg, B.—(1937) *Hospitalstidende*, **80**, 881.
Horton, B. T.—(1928) *Amer. J. Anat.*, **41**, 197.
Jamieson, J. K., and Dobson, J. F.—(1907) *Lancet*, i, 1061.
Kocher, T.—(1893) *KorrespondBl. schweiz. Arz.*, **23**, 682.
Loewy, G., and Bertrand, I.—(1939) *Arch. Mal. Appar. dig.*, **29**, 407.
Mikulicz, J.—(1898) *Arch. klin. Chir.*, **57**, 254.
Most, A.—(1899) *Ibid.*, **59**, 175.
Moynihan, B.—(1926) 'Abdominal Operations,' vol. i. London (Saunders), p. 384.
Rokitansky, C.—(1861). Quoted by Brinton (1864).
Sherren, J.—(1932) 'System of Surgery (Choyce),' vol. ii, 3rd ed. London (Cassell), p. 280.
Stewart, M. J.—(1947) *Brit. J. Radiol.*, **20**, 505.
Walters, W., Gray, H. K., and Priestley, J. T.—(1942) 'Carcinoma and other Malignant Diseases of the Stomach.' Philadelphia (Saunders), p. 309.
Willis, R. A.—(1948) 'Pathology of Tumours.' London (Butterworth), p. 406.